# Hypercoagulation Detected by Rotational Thromboelastometry Predicts Mortality in COVID-19: A Risk Model Based on a Prospective Observational Study

**DOI:** 10.1055/a-1725-9221

**Published:** 2022-03-07

**Authors:** Lou M. Almskog, Agneta Wikman, Jonas Svensson, Matteo Bottai, Mariann Kotormán, Carl-Magnus Wahlgren, Michael Wanecek, Jan van der Linden, Anna Ågren

**Affiliations:** 1 Department of Anaesthesiology and Intensive Care, Capio St. Göran's Hospital, Stockholm, Sweden; 2 Department of Molecular Medicine and Surgery, Karolinska Institutet, Stockholm, Sweden; 3 Department of Clinical Immunology and Transfusion Medicine, Karolinska University Hospital, Stockholm, Sweden; 4 Department of CLINTEC, Karolinska Institutet, Stockholm, Sweden; 5 Department of Clinical Neuroscience, Centre for Psychiatry Research, Karolinska Institutet & Stockholm Health Care Services, Region Stockholm, Karolinska University Hospital, Stockholm, Sweden; 6 Division of Biostatistics, Institute of Environmental Medicine, Karolinska Institutet, Stockholm, Sweden; 7 Department of Vascular Surgery, Karolinska University Hospital, Stockholm, Sweden; 8 Department of Physiology and Pharmacology, Karolinska Institutet, Stockholm, Sweden; 9 Perioperative Medicine and Intensive Care, Karolinska University Hospital, Stockholm, Sweden; 10 Coagulation Unit, Hematology Centre, Karolinska University Hospital, Stockholm, Sweden; 11 Department of Clinical Sciences, Danderyd Hospital and Karolinska Institutet, Stockholm, Sweden

**Keywords:** COVID-19, thromboelastometry, coagulopathy, thrombosis, mortality

## Abstract

**Background** Severe disease due to the novel coronavirus disease 2019 (COVID-19) has been shown to be associated with hypercoagulation. The aim of this study was to assess the Rotational Thromboelastometry (ROTEM) as a marker of coagulopathy in hospitalized COVID-19 patients.

**Methods** This was a prospective, observational study where patients hospitalized due to a COVID-19 infection were eligible for inclusion. Conventional coagulation tests and ROTEM were taken after hospital admission, and patients were followed for 30 days. A prediction model, including variables ROTEM EXTEM-MCF (Maximum Clot Firmness) which in previous data has been suggested a suitable marker of hypercoagulation, age, and respiratory frequency, was developed using logistic regression to evaluate the probability of death.

**Results** Out of the 141 patients included, 18 (13%) died within 30 days. In the final prediction model, the risk of death within 30 days for a patient hospitalized due to COVID-19 was increased with increased EXTEM-MCF, age, and respiratory frequency. Longitudinal ROTEM data in the severely ill subpopulation showed enhanced hypercoagulation. In an in vitro analysis, no heparin effect on EXTEM–coagulation time (CT) was observed, supporting a severe acute respiratory syndrome-coronavirus-2 (SARS-CoV-2) effect on prolonged initiation of coagulation.

**Conclusion** Here, we show that hypercoagulation measured with ROTEM predicts 30-day mortality in COVID-19. Longitudinal ROTEM data strengthen the hypothesis of hypercoagulation as a driver of severe disease in COVID-19. Thus, ROTEM may be a useful tool to assess disease severity in COVID-19 and could potentially guide anticoagulation therapy.

## Introduction

The global emergence of the novel corona virus disease 2019 (COVID-19), caused by the severe acute respiratory syndrome-coronavirus 2 (SARS-CoV-2), has evolved rapidly achieving pandemic proportions with dire consequences for human health and welfare. 1 Though several risk factors for severe disease are known (e.g., high age, obesity, diabetes, and chronic pulmonary disease), 2 there is still a need of prognostic models focusing on identifying patients at high risk of death.

Recent reports indicate a high incidence of thrombotic events in COVID-19 patients treated in intensive care units (ICUs), 3 even in patients receiving therapeutic anticoagulation. 4 This suggests that an essential pathophysiological component of COVID-19 is related to a widespread and persistent hypercoagulation 5 where the systemic inflammation induced by SARS-CoV-2 activates the coagulation systems, 6 provoking proinflammatory cytokines 7 causing in situ thrombosis. 8 The underlying mechanisms of the prothrombotic state remain to be clarified. 9

Elevated levels of fibrin degradation products (e.g., D-dimer) have consistently been suggested as a strong prognostic factor associated with poor outcome, 10 and early identification of patients at risk of developing thromboembolic complications due to COVID-19 infection may contribute to more adequate antithrombotic strategies. Rotational Thromboelastometry (ROTEM) is a clinically well-established blood test, used for monitoring coagulopathy, 11 12 also in cases where conventional coagulation tests (CCTs) may fail, 13 14 limited by their inability to assess clot strength, fibrinogen functionality, and fibrinolysis. 15 The possibility to detect and quantify hypercoagulative states is an important advantage of ROTEM compared with CCTs. 13 16 Furthermore, ROTEM variables may be affected earlier during the disease course in COVID-19 compared with other markers (e.g., D-dimer) 17 and may therefore be of greater value as predictors of adverse outcome.

Previous studies assessing ROTEM in critically ill COVID-19 patients suggest a procoagulant state 18 19 and this pattern has also been observed in earlier stages of the COVID-19 disease. However, a prolonged EXTEM coagulation time (EXTEM-CT), more pronounced in patients at higher care levels, indicates a prolonged initiation of coagulation in COVID-19. 20

Several risk stratification tools referring to patients with COVID-19 across different settings and populations have been reported 2 and ROTEM in combination with D-dimer have been verified to predict thromboembolic risks in COVID-19. 4 However, no data evaluating ROTEM as a predictor of mortality have, to the best of our knowledge, yet been published.

In this study, we aimed to evaluate several markers of coagulopathy in patients hospitalized due to COVID-19. Specifically, we intended to:

- Develop and test a pragmatic risk stratification score model, using ROTEM data in combination with other known risk factors to predict 30-day mortality.
- Assess the longitudinal course of ROTEM test results in severe disease.
- Examine the low molecular weight heparin (LMWH) effect detected by ROTEM, analyzed in an in vitro experiment.
- Evaluate the D-dimer-to-P-fibrinogen ratio as a marker of thrombotic activity.

## Methods

### Study Design

The study was a prospective, observational single-center study. Inclusion criteria were hospitalization due to verified COVID-19 infection and age over 18 years. No exclusion criteria were set up. After inclusion, a blood sample was taken and ROTEM analyzed, apart from this nothing differed from the standard care. The ROTEM analyses in this study were performed for research purpose only and ROTEM test results, as opposed to other laboratory test results, were not available for the treating physician and did therefore not affect treatment. All patients were followed-up after 30 days when outcomes were registered. The study was approved by the Swedish Ethical Review Authority (D-nr 2020–01875). In this ethical approval, consent was waived in very severe cases of COVID-19 disease where patients, due to medical conditions, were not able to give their consent.

### Study Population

All patients enrolled in the study were included at Capio St Göran's Hospital, Stockholm, Sweden, during a 4-month recruitment period (May–August 2020). The diagnosis of COVID-19 was defined as either positive polymerase chain reaction (PCR) test for SARS-CoV-2, or as typical radiologic findings on chest computed tomography (CT), corresponding to COVID-19 classification score (CO-RADS) 4 or 5. 21 Patients were admitted either to regular wards with possibility of low-flow oxygen therapy, or to intermediate wards with more advanced ventilation support; noninvasive ventilation (NIV) or nasal high-flow (NHF) oxygen therapy or to the intensive care unit (ICU) where, in addition to NIV/NHF, invasive mechanical ventilation support were available. Extracorporeal membrane oxygenation (ECMO) is not available at Capio St Göran's Hospital.

### Definitions

In the statistical model, the ROTEM variable EXTEM-MCF (Maximum Clot Firmness) was deemed the most suitable candidate as a marker of hypercoagulation, based on a previously published analysis of a subset of the data. 20

Comorbidity in the statistical model was defined as a prior diagnosis of either hypertension, diabetes, chronic obstructive pulmonary disease (COPD)/asthma, or cardiovascular disease.

Previous thromboembolic disease among included patients was defined as a diagnosis of either arterial or venous thrombosis, registered in the medical journal at any time, prior to presentation of COVID-19 symptoms.

### Laboratory Testing

ROTEM and CCTs (D-dimer, P-fibrinogen, activated partial thromboplastin time (APTT), the International Normalized Ratio (INR), antithrombin, and platelet count) were collected after admission (hospital median day 2, [interquartile range (IQR): 1–3]). Apart from these blood tests, nothing in the COVID-19 standard care of included patients was changed including ventilation strategies, medications, or routine examinations. To examine the longitudinal course of ROTEM in more severely ill patients, we performed repeated testing at days 5 and 10 after the first blood test. Patients who were discharged from hospital care prior to the second or third test were not tested. This longitudinal sample will therefore represent the development over time in cases with a more severe disease course. Furthermore, the D-dimer-to-P-fibrinogen ratio was calculated as a marker of thrombotic activity.

### Anticoagulant Therapy

During the study period, anticoagulant therapy was standard of care in COVID-19 pneumonia at Capio St Göran's hospital and was prescribed according to disease severity and thromboembolic risk profile. Routine anticoagulant treatment administrated after admission was the LMWH Tinzaparin (Innohep, LEO Pharma, Copenhagen, Denmark) classified after dose regime (low prophylaxis dose [75 IE/kg/24 h], high prophylaxis dose [150 IE/kg/24 h], or treatment dose [≥175 IE/kg/24 h]). Patients with severe disease requiring ventilation support received high prophylaxis doses of antithrombotic treatment, and in case of verified or suspected thromboembolic disease, treatment doses of LMWH were used. Patients in regular wards without risk factors of thromboembolic complications received low-dose prophylaxis.

### Rotational Thromboelastometry Analysis

ROTEM is an established point-of-care device, used for detecting and monitoring coagulopathy, providing rapid assessment of clot formation to lysis. A ROTEM sigma (Tem Innovations GmbH, Germany) was used for thromboelastometric analyses. Here, we present four ROTEM-variables as follows: (1) extrinsically activated assays with tissue factor (EXTEM), (2); intrinsically activated assays using phospholipid and ellagic acid (INTEM), (3) fibrin-based extrinsically activated assays with tissue factor and platelet inhibitor cytochalasin D (FIBTEM), and (4) intrinsically activated assays with the addition of heparinase (HEPTEM). EXTEM and INTEM test the extrinsic and intrinsic pathways, respectively. FIBTEM provides information of fibrinogen function, eliminating platelet contribution to clot formation. HEPTEM eliminates heparin effects. Within every ROTEM-variable, five parameters were quantified: CT which is the time (in seconds) from test start until an amplitude of 2 mm is reached, giving information about coagulation activation/initiation. Clot formation time (CFT) corresponds to the time (in seconds) between 2- and 20-mm amplitudes, giving information about clot propagation. Maximum clot firmness (MCF) is the maximum amplitude (in mm) reached during the test, giving information about clot stability. Lysis index (LI) 30 and 60 are the reduction in MCF 30 and 60 minutes after CT, respectively (in percent). 22

A prolonged EXTEM-CT, short EXTEM-CFT, and an increased EXTEM-MCF and/or FIBTEM-MCF suggest a hypercoagulable state with a prolonged initiation of coagulation. A prolonged INTEM-CT compared with HEPTEM-CT illustrates a heparin effect. To test the effects of different doses of tinzaparin on EXTEM-CT in vitro, four different concentrations of tinzaparin were added to blood from healthy donors ( *n* = 3), and we applied a repeated measures of analysis of variance (ANOVA).

### Statistical Analysis

Considering the relatively small sample size, the predictors to be included in the prediction model were chosen based on a priori knowledge. 2 These were age, MCF, and RF.

Categorical variables were introduced in the regression model by means of dummy variables. Numeric covariates were transformed with the most suitable power transformation. The transformed variables were entered to the predictive models through natural cubic splines when significant departures from linearity were detected. The choice of number and location of the knots were based on visual assessment and the Akeike's information criterion, respectively. The predictive properties of the model were evaluated by calculating the area under the curve (AUC) for the receiver operating characteristic (ROC) curve. Sensitivity, specificity, and positive and negative predictive values were calculated. All continuous variables were presented as median and IQR. Two-sided Wilcoxon's test was used to test for difference between groups for continuous variables and Fisher's exact test for categorical data. In the longitudinal data analysis, we used two-sided, paired Wilcoxon's test for the ROTEM variables EXTEM-CT, -MCF, and -CFT, respectively. *p* -Values below 0.05 were considered statistically significant. Stata statistical software, version 15 (StataCorp LLC) and R, version 3.6.1 was used for statistical analysis and visualizations.

This study was conducted and reported applying the Strengthening the Reporting of Observational Studies in Epidemiology (STROBE) guidelines.

## Results

### Demographic and Clinical Characteristics

A total of 141 COVID-19 patients with a median age of 63 years [IQR: 51–75] were included in the study, 87 (62%) were male ( Table 1 ). Comorbidities were common; 45% of patients had a prior diagnosis of hypertension, 24% diabetes, 26% other chronic diseases (renal failure, rheumatological or neurological disease), and 28 patients (20%) had previous thromboembolic disease.

**Table 1:** Baseline characteristics: survivors and nonsurvivors

	Total *n* (%)/median (IQR)	Survivors *n* (%)/median (IQR)	Nonsurvivors *n* (%)/median (IQR)	*p* -Value
	*n* = 141	*n* = 123	*n* = 18	
Gender male	87 (62)	74 (60)	13 (72)	0.44
Age (y)	63 (51–75)	61 (49–74)	74 (66–80)	0.002
BMI (kg/m ^2^ )	27 (24–31)	26 (24–30)	29 (26–33)	0.04
Previous thromboembolic disease	28 (20)	19 (15)	9 (50)	0.002
Antithrombotic treatment at inclusion	25 (18)	16 (13)	9 (50)	<0.001
Diabetes	34 (24)	26 (21)	8 (44)	0.04
Smoking	15 (11)	13 (11)	2 (11)	1
COPD/asthma	20 (14)	18 (15)	2 (11)	1
Hypertension	63 (45)	50 (41)	13 (72)	0.02
Cardiovascular disease	29 (21)	19 (15)	10 (56)	<0.001
Malignancy	18 (13)	15 (12)	3 (17)	0.7
Other diseases	37 (26)	31 (25)	6 (33)	0.57
Respiratory frequency at inclusion (breaths/min)	20 (18–24)	20 (18–24)	26 (24–30)	<0.001
Saturation at inclusion (%)	95 (93–98)	96 (93–98)	92 (90–94)	0.002
Days with COVID-19 symptoms at inclusion	10 (7–14)	9 (7–14)	11 (9–14)	0.36
Total hospital days at inclusion	2 (1–3)	2 (1–2)	3 (2–8)	0.003
Thrombosis at inclusion	7 (5)	6 (5)	1 (6)	1
Thrombosis after inclusion	8 (6)	6 (5)	2 (11)	0.27
Anticoagulant prophylaxis before ROTEM analysis	101 (72)	85 (69)	16 (89)	0.1

Abbreviations: BMI, body mass index; COPD, chronic obstructive pulmonary disease; COVID-19, novel corona virus disease 2019; IQR, interquartile range; ROTEM, Rotational Thromboelastometry.

Demographic and clinical baseline characteristics for all patients were divided in survivors and nonsurvivors ( Table 1 ). Eighteen patients (13%) died within 30 days after inclusion (“nonsurvivors”). Nonsurvivors were older and had higher body mass index (BMI) than survivors. Further, among nonsurvivors, a larger proportion of patients had previous thromboembolic disease, diabetes, and hypertension compared with survivors. Altogether, these observations among nonsurvivors reflect a more pronounced disease burden at baseline, compared with survivors. Nonsurvivors also had higher respiratory frequency and lower blood oxygen saturation at inclusion compared with survivors. Number of days with COVID-19 symptoms prior to inclusion did not differ significantly between survivors and nonsurvivors. Eight patients (6%) had thrombosis during hospitalization (two pulmonary emboli, three myocardial infarctions, and three distal venous thrombosis).

Among nonsurvivors, 9 of 18 patients (50%) had ongoing anticoagulant or platelet inhibiting treatment before hospital admission, prescribed prior to their SARS-CoV-2 infection (five patients were treated with direct oral anticoagulants [DOAC], one patient with warfarin, one patient with LMWH, and two patients with platelet inhibitors). Ten (56%) of nonsurvivors had cardiovascular disease (e.g., atrial fibrillation and ischemic heart disease). Among survivors, 16 of 123 (13%) had anticoagulant treatment prescribed before inclusion and 19 (15%) had a history of cardiovascular disease.

### Laboratory Test Results

Laboratory test results are presented in Table 2 . D-dimer and APTT were significantly increased in nonsurvivors compared with survivors ( *p* = 0.01 and 0.002). Platelet count, INR, P-fibrinogen, and antithrombin did not differ significantly among survivors compared with nonsurvivors. EXTEM-/INTEM-CT were significantly prolonged in non-survivors compared with survivors. EXTEM-/FIBTEM-MCF were in upper reference ranges and EXTEM-/INTEM-LI30 and -LI60 indicated low fibrinolytic activity in survivors, as well as nonsurvivors.

**Table 2:** Laboratory test results at inclusion; survivors and non-survivors

	Total Median (IQR)	Survivors Median (IQR)	Nonsurvivors Median (IQR)	*p* -Value	Reference range
	*n* = 141	*n* = 123	*n* = 18		
COVID-19 positive	141 (100%)	123 (100%)	18 (100%)	–	
D-dimer (mg/L)	0.7 (0.5–1.4)	0.7 (0.5–1.3)	1.6 (0.6–3.0)	0.01	<0.5
P-fibrinogen (g/L)	5.5 (4.4–6.9)	5.4 (4.3–6.9)	5.9 (5.3–7.1)	0.14	1.8–3.8
D-dimer-to-P-fibrinogen ratio (%)	0.015 (0–0.030)	0.014 (0.001–0.028)	0.021 (0.001–0.042)	0.16	
APTT (s)	26 (24–30)	26 (24–29)	31 (27–33)	0.002	24–32
INR	1.0 (1.0–1.1)	1.0 (1.0–1.1)	1.1 (1.0–1.4)	0.15	0.9–1.2
Antithrombin (kIE/L)	1.0 (0.9–1.1)	1.0 (0.9–1.1)	0.9 (0.9–1.1)	0.18	0.8–1.2
Platelet count (10 ^9^ /L)	225 (169–291)	222 (170–289)	243 (163–345)	0.49	Men: 145–348, women: 165–387
Hemoglobin (g/L)	129 (115–140)	119 (111–127)	128 (115–139)	0.04	Men: 130–170, women: 120–155
White blood cell count (10 ^9^ /L)	6.5 (5.0–9.3)	6.4 (4.6–9.1)	8.0 (6.2–10.8)	0.08	3.5–8.8
Creatinine (μmol/L)	63 (54–79)	83 (63–106)	64 (54–83)	0.02	Men: 60–105, women: 45–90
EXTEM-CT (s)	71 (61–87)	70 (61–84)	94 (78–146)	<0.001	38–79
EXTEM-CFT (s)	49 (41–61)	49 (42–60)	51 (42–69)	0.44	34–159
EXTEM-MCF (mm)	71 (67–75)	70 (67–74)	74 (69–77)	0.05	50–72
EXTEM-LI30 (%)	100 (100–100)	100 (100–100)	100 (100–100)	–	94–100
EXTEM-LI60 (%)	97 (96–98)	97 (95–99)	98 (98–99)	0.05	90–92
INTEM-CT (s)	185 (170–199)	183 (170–195)	200 (192–216)	0.003	100–240
INTEM-CFT (s)	55 (44–69)	55 (44–70)	54 (40–63)	0.41	30–110
INTEM-MCF (mm)	67 (63–72)	67 (63–72)	73 (67–74)	0.02	50–72
INTEM-LI30 (%)	100 (100–100)	100 (100–100)	100 (100–100)	–	94–100
INTEM-LI60 (%)	98 (96–100)	97 (95–99)	99 (99–99)	0.05	90–93
FIBTEM-MCF (mm)	29 (24–34)	28 (24–33)	30 (28–37)	0.09	9–25
HEPTEM-CT (s)	185 (174–198)	182 (174–196)	204 (183–213)	0.01	100–240

Abbreviations: APTT, activated partial thromboplastin time; CFT, clot formation time; CT, coagulation time; EXTEM, extrinsically activated assays with tissue factor; FIBTEM, fibrin-based extrinsically activated assays with tissue factor and platelet inhibitor cytochalasin D; HEPTEM, Intrinsically activated assays with the addition of heparinase; INR, International Normalized Ratio; INTEM, intrinsically activated assays using phospholipid and ellagic acid; IQR, interquartile Range; LI-30, lysis index-30; LI-60, lysis index-60; MCF, maximum clot firmness.

### Prediction Model

The final logistic regression prediction model:

*Logit* ( *death* ) = −9.6 + (9.5) * MCF + (0.15) * age + (−155) * RF

where *Logit* ( *death* ) is the predicted log odds of 30-day mortality (here synonymous with the risk score); MCF is the cubic power of EXTEM-MCF divided by 10 ^6^ ; age is in years and not transformed; RF is the reciprocal (1/ *x* ) of the respiratory frequency at inclusion.

According to this model, the risk of death within 30 days for a patient hospitalized due to COVID-19 was increased with increased age, respiratory frequency, and EXTEM-MCF (all predictors *p* < 0.05; Table 3 ).

**Table 3:** Logistic regression for mortality at 30 days

	Coefficient	Standard error	*z*	*p* > ∣ *z* ∣	95% confidence interval	
EXTEM-MCF (mm)	9.49	4.65	2.04	0.041	0.37	20
Respiratory frequency (breaths/min)	−154.97	41.54	−3.73	0.000	−236.38	−73.56
Age (y)	0.15	0.047	3.27	0.001	0.061	0.25
Constant	−9.59	3.89	−2.47	0.014	−17.22	−1.97

Abbreviations: EXTEM, extrinsically activated assays with tissue factor; MCF, maximum clot firmness.

**Fig. 1:**
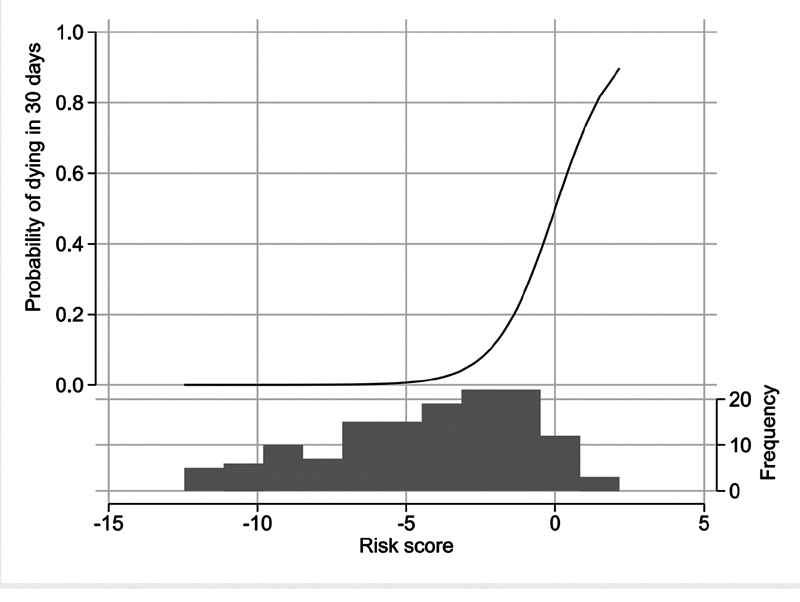
Predicted probability of death vs risk score. Distribution of patients across range of risk scores, corresponding to the logit (logged odds ratio) calculated using logistical regression with the three predictor variables at inclusion (EXTEM-MCF, age, and respiratory frequency). The histogram in black, presented in the lower part of the figure, shows the distribution of risk scores in the present data (e.g., 12 patients have a risk score of 0, translating to approximately 50% probability of dying within 30 days). MCF, maximum clot firmness.

The risk score generated by the model may be transformed to the more intuitive variable probability of death ( Fig. 1 ).

Through changing one predictor while keeping other variables constant, it is possible to illustrate the effect of the predictors on the probability of death. If, for example, respiratory frequency is kept constant at 20 breaths/min (median value in the full sample), as EXTEM-MCF is increased from 65 to 75, mortality risk increases from 0.1 to 0.4% in a 51-year-old patient (lower age quartile), and from 3.8 to 13.7% in a 75-year-old (higher age quartile; Table 4 ).

**Table 4:** Mortality 30 days in %, related to age (y) and EXTEM-MCF (mm)

	EXTEM-MCF 65	EXTEM-MCF 70	EXTEM-MCF 75
Age = 51 years, RF = 20	0.1%	0.2%	0.4%
Age = 63 years, RF = 20	0.6%	1.2%	2.5%
Age = 75 years, RF = 20	3.8%	7.0%	13.7%

Abbreviations: MCF, maximum clot firmness; RF, respiratory frequency.

When the model is applied to the data, the ROC curve AUC is 0.91. If the cut-off for probability of death is set to 0.13, this corresponds to a sensitivity of 94%, specificity of 81%, a positive predictive value of 41%, and a negative predictive value of 99%.

### Longitudinal Data

**Fig. 2:**
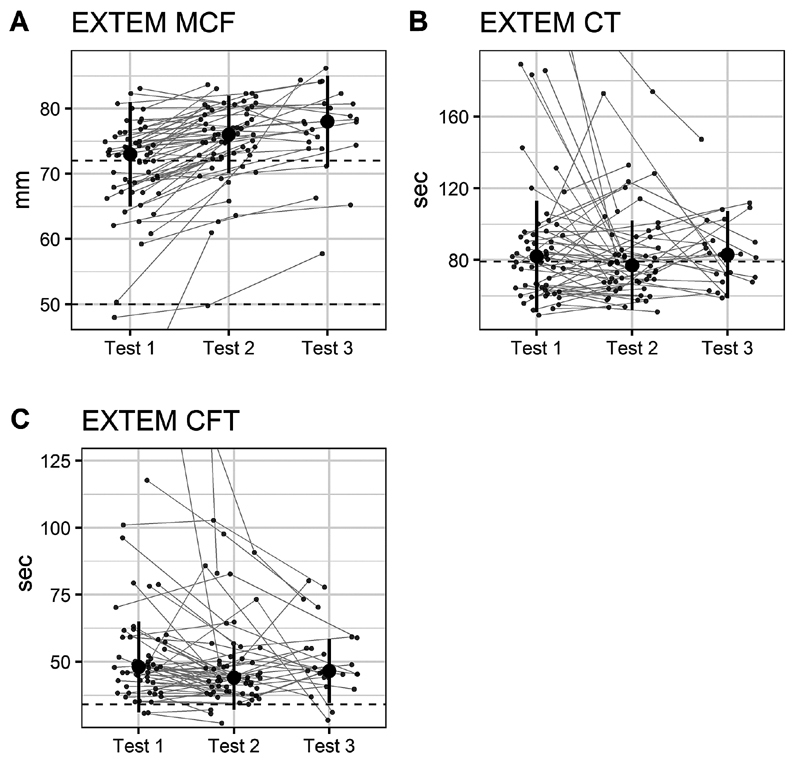
Longitudinal ROTEM data. ROTEM sampled at inclusion, after 5 days and after 10 days. In ( **A** ) EXTEM- MCF (dashed horizontal lines are upper and lower reference values: 50–72 mm); ( **B** ) EXTEM-CT (dashed horizontal line is upper reference value: 79 second); ( **C** ) EXTEM-CFT (dashed horizontal line is lower reference value: 34 second). Median values reported with error bars representing IQR. For visualization purposes some outliers are not shown but are represented by lines connecting them to follow up measurements. Abbreviations: CFT, clot formation time; CT, coagulation time; EXTEM, extrinsically activated assays with tissue factor; IQR, interquartile range; MCF, maximum clot firmness; ROTEM, Rotational Thromboelastometry.

In the longitudinal analysis, 57 patients were tested a second time, and of these 24 patients were tested a third time. EXTEM-MCF increased from median 73 [IQR: 65–81] to 76 mm [IQR: 68–84] with *p* < 0.001. For the subset of patients tested a third time, the value increased from 75.5 [IQR: 68–83] to 78 mm [IQR 71–85] with *p* = 0.006. EXTEM-CFT decreased from median 48 [IQR: 31–65] to 44 seconds [IQR 32–56] with *p* < 0.001. For the subset of patients tested a third time the second test was 49 seconds [IQR 27–71] and the third test was 46.5 seconds [IQR 34.5–58.5] with *p* = 0.13. No significant changes in EXTEM-CT were observed, first and second test medians were 82 [IQR 51–113] and 77 seconds [IQR 52–102] with *p* = 0.19. For the subset tested a third time, the second test was 84 seconds [IQR 55–113] and the third test was 83 seconds [IQR 59–107] with *p* = 0.42 ( Fig. 2 ).

### INTEM-HEPTEM Difference

To assess the heparin effect on our test results, we evaluated the difference between INTEM-CT and HEPTEM-CT in all patients receiving LMWH before inclusion ( *n* = 86). In this subgroup, we did not observe any significant INTEM-HEPTEM CT difference ( *p* = 0.55) indicating no heparin effect on our ROTEM results.

### In Vitro Analysis

In our dataset, we observed a prolongation of EXTEM-CT, more pronounced in nonsurvivors compared with survivors. A majority of patients in both groups had received LMWH prior to inclusion (nonsurvivors, 89% and survivors 69%). Previous in vitro data have shown no effect on EXTEM-CT by LMWH (Dalteparin/Fragmin, Pfizer, New York, United States) in therapeutic doses. 23 To determine whether prolonged EXTEM-CT may possibly be associated with higher doses of LMWH, we performed an experimental study with tinzaparin in vitro ( Table 5 ) where increasing doses of tinzaparin did not result in a statistically significant change of EXTEM-CT ( *p* = 0.6, repeated measures ANOVA).

**Table 5:** INTEM-/EXTEM-/HEPTEM-CT at different tinzaparin concentrations, where 1.0 IU/mL corresponds to a tinzaparin dose of 4,500 IU

	Tinzaparin concentration (IU/mL)				
	0	0.5	1.0	1.5	2.0
Donor 1					
INTEM-CT	170	253	368	220	332
EXTEM-CT	43	56	48	49	52
HEPTEM-CT	172	139	239	148	165
Donor 2					
INTEM-CT	231	269	358	447	453
EXTEM-CT	67	60	56	57	60
HEPTEM-CT	172	180	195	197	218
Donor 3					
INTEM-CT	264	353	282	338	348
EXTEM-CT	50	47	49	43	52
HEPTEM-CT	270	131	249	167	294

Abbreviations: CT, coagulation time; EXTEM, extrinsically activated assays with tissue factor; HEPTEM, intrinsically activated assays with the addition of heparinase; INTEM, intrinsically activated assays using phospholipid and ellagic acid.

### D-Dimer-to-P-Fibrinogen Ratio

The D-dimer-to-P-fibrinogen ratio reflects fibrinolysis in relation to fibrin deposition and has previously been defined as a marker of thrombotic activity, where a higher ratio correlates to a more thrombogenic profile. 24 In our data, an increased ratio was observed in nonsurvivors compared with survivors, 0.021 [IQR: 0.001–0.042] and 0.014 [IQR: 0.001–0.028], respectively ( Table 2 ). The difference was not statistically significant ( *p* = 0.16).

## Discussion

This study reports a stratification risk score model where we evaluate the ability of ROTEM to predict mortality in COVID-19 patients. Our results support the concept of an early pronounced hypercoagulability, measured by increased EXTEM-MCF on admission that is associated with an increased mortality risk. In combination with age and respiratory frequency, which are two easily measured clinical parameters, our model introduces a feasible tool to assess the risk of death in COVID-19 pneumonia.

There is an abundance of findings suggesting that hypercoagulopathy is a crucial component in the pathophysiology of severe COVID-19. Importantly, enhanced anticoagulant treatment has been shown to be associated with reduced mortality, 25 as well as a reduction in inflammatory biomarkers. 26

The covariates included in the model (age, respiratory frequency) have in earlier studies been suggested as important predictors of clinical outcome. High age is one of the most frequently reported predictors of poor prognosis in COVID-19 and respiratory frequency has in patients with COVID-19 been described as a predictor of mechanical ventilation and in-hospital mortality. 2 EXTEM-MCF is a suitable indicator of clot stability and commonly used in a clinical context 27 which made us choose this variable for our prediction model. When these three predictors were modeled, we observed a high sensitivity of 94% and a high specificity of 81% in our data.

In our patients, we typically found prolonged initiation of coagulation (prolonged EXTEM-/INTEM-CT), shortened clot propagation (shortened EXTEM-CFT) with a subsequent forming of a very stable clot with a pronounced clot firmness (increased EXTEM-/FIBTEM-MCF) and coexisting hypofibrinolysis (increased EXTEM-/INTEM-LI60). Our longitudinal analysis supports these findings, where increasing EXTEM-MCF and shortening of EXTEM-CFT were observed confirming enhanced hypercoagulation.

Prolonged values of EXTEM-/INTEM-CT are, in contrast to other ROTEM variables, not an indication of hypercoagulation. Increased INTEM-CT in nonsurvivors may be due to anticoagulant treatment effects. Increased EXTEM-CT is in line with previous studies where prolongation of prothrombin time with similar activation proteins as in EXTEM was identified in COVID-19 patients. 28 29 In our in vitro analysis results, no impact on EXTEM-CT was observed with increasing LMWH doses, supporting the hypothesis that prolonged initiation of coagulation in COVID-19 may be due to viral effects and inflammatory activation.

Low fibrinolytic activity was observed in survivors, as well as nonsurvivors in our data, indicating hypofibrinolysis/fibrinolysis shutdown in both EXTEM and INTEM. 30 Decreased fibrinolysis may partially explain the hypercoagulability observed in COVID-19. Indeed, earlier reports suggest that elevated levels of plasminogen activator inhibitor type 1 (PAI-1), which is one of the most important inhibitors of the fibrinolytic system, may result in lower plasmin activity and hence decreased fibrinolysis. This in turn, may contribute to an imbalance between coagulation and fibrinolysis in COVID-19. 31

Increased levels of P-fibrinogen, corresponding to high FIBTEM-MCF values, indicate pronounced clot stability and are shown to be characteristic for COVID-19 hypercoagulation. 32 However, in previous data, the correlation between hypercoagulation and FIBTEM has been reported even stronger than the correlation to EXTEM. 33 Both FIBTEM and EXTEM are extrinsically activated assays with tissue factor, though in FIBTEM, platelets are inhibited by cytochalasin D. 22 Consequently, when evaluating clot stability in vivo, we found EXTEM to be more informative compared with FIBTEM, as the contribution of platelets to hypercoagulation is taken into consideration.

We observed elevated levels of P-fibrinogen and D-dimer in our data which is in line with previous studies on COVID-19 and coagulopathy. 7 34 However, these variables are considered acute inflammatory plasma markers expected to rise during inflammation, and neither parameter has been shown to reliably identify patients with increased thromboembolic risks in COVID-19. 7 Furthermore, the D-dimer increase is not always evident in early stages of the disease limiting its usefulness as a prognostic tool. 29

The D-dimer-to-P-fibrinogen ratio is an indicator of prothrombotic activity where a higher ratio suggests a more pronounced thrombotic state. An increased ratio in COVID-19 may reflect the presence of activated coagulation leading to fibrinogen consumption in the pulmonary vasculature, with simultaneous activation of fibrinolysis resulting in elevated D-dimer levels. 35 In our data, we observed a higher D-dimer-to-P-fibrinogen ratio in nonsurvivors compared with survivors but the difference was not significant.

## Limitations and Strengths

Some limitations of this study should be recognized. First, the sample size of 141 patients (of which 18 died within 30 days) was relatively small, given the goal of developing a prediction model. To avoid overfitting, we were limited in the number of predictor variables we evaluated. Second, the data were collected at a single site, and the prediction model was not validated in an independent sample, limiting the generalizability of our findings in other settings and populations. As the data collection was made in the beginning of the SARS-CoV-2 pandemic, no clinical COVID-19 guidelines were yet available, neither were published patient outcome data to compare with, limiting the possibilities of performing external validation at that point. Third, some patients were included somewhat later than the day of admission, which may have reflected test results of different disease stages. Fourth, most patients had received antithrombotic treatment prior to inclusion which may have influenced our laboratory results. However, given that the ROTEM variable we chose to include as a predictor indicates hypercoagulopathy, we do not presume this created any false positive associations.

These limitations notwithstanding, we consider our cohort as a representative sample from the first wave of COVID-19 in Stockholm in which a state of hypercoagulability has been shown to be associated with an increased risk of death. Together, these results indicate that ROTEM is a useful analyzing method of coagulopathy in COVID-19 and may be a promising tool to guide anticoagulant treatment.

## Conclusion

In this study, we evaluated ROTEM as a marker of coagulopathy in COVID-19. First, we presented a risk stratification score model where increased EXTEM-MCF, in combination with age and respiratory frequency, was predictive of increased mortality within 30 days. We then assessed the longitudinal disease course where ROTEM supported the hypothesis of enhanced hypercoagulation in severe disease. Our data did not indicate any effect on EXTEM-CT with increasing LMWH concentrations. In conclusion, ROTEM may be helpful to tailor anticoagulant therapy and suggested as a feasible tool for monitoring disease, which may improve survival in patients with a poor prognosis in COVID-19.

## List of Abbreviations

Rotational Thromboelastmetry (ROTEM)
Extrinsically activated assays with tissue factor (EXTEM)
Maximum Clot Firmness (MCF)
Activated Partial Thromboplastin Time (APTT)
Coagulation Time (CT)
Intrinsically activated assays using phospholipid and ellagic acid (INTEM)
Corona virus disease 2019 (COVID-19)
Severe acute respiratory syndrome coronavirus 2 (SARS-CoV-2)
Intensive Care Unit (ICU)
Conventional coagulation test (CCT)
Polymerase chain reaction (PCR)
Computed tomography (CT)
Non-invasive ventilation (NIV)
Nasal high flow oxygen therapy (NHF)
Extracorporeal Membrane Oxygenation (ECMO)
Chronic obstructive pulmonary disease (COPD)
International Normalized Ratio (INR)
Low Molecular Weight Heparin (LMWH)
Fibrin-based extrinsically activated assays with tissue factor and platelet inhibitor cytochalasin D (FIBTEM)
Intrinsically activated assays with the addition of heparinase (HEPTEM)
Clot Formation Time (CFT)
Lysis Index (LI)
Body mass index (BMI)
Area under the curve (AUC)
Receiver operating characteristic (ROC)
Interquartile range (IQR)
Strengthening the Reporting of Observational Studies in Epidemiology (STROBE)
Direct oral anticoagulants (DOAC)
Respiratory frequency (RF)

## Notes

**What Is Known about This Topic?**

- A high prevalence of thromboembolic complications is reported in the novel coronavirus disease 2019 (COVID-19).
- Rotational Thromboelastometry (ROTEM) is a well-established method for monitoring coagulopathic conditions.
- Several risk factors have been shown to predict a severe disease course in COVID-19, but prognostic models incorporating data on hypercoagulation are lacking.

**What Does This Paper Add?**

- We present baseline and follow-up data of 141 patients in a large hospital in Stockholm, Sweden, during the first wave of COVID-19.
- We have developed a prognostic model using logistic regression, where hypercoagulation detected by ROTEM predicts mortality in COVID-19 disease.
- We present longitudinal data confirming hypercoagulation as a driver of severe disease in COVID-19.
